# Music therapy in artificial insemination by husband: Effects on psychological distress and clinical pregnancy rate

**DOI:** 10.1097/MD.0000000000050655

**Published:** 2026-09-11

**Authors:** Xianwen Jin, Rubing Hu, Xiaocha Zhu, Xuewei Chen

**Affiliations:** a Department of Reproductive Outpatient Care, Center for Reproductive, The Fourth Affiliated Hospital of School of Medicine, and International School of Medicine, International Institutes of Medicine, Yiwu, Zhejiang Province, China; b Department of Reproductive, Center for Reproductive, The Fourth Affiliated Hospital of School of Medicine, and International School of Medicine, International Institutes of Medicine, Yiwu, Zhejiang Province, China; c Department of Comprehensive Ward, The Fourth Affiliated Hospital of School of Medicine, and International School of Medicine, International Institutes of Medicine, Yiwu, Zhejiang Province, China.

**Keywords:** artificial insemination by husband, clinical pregnancy rate, Depression Anxiety Stress Scales-21, infertility, music therapy, psychological distress

## Abstract

**Background:**

Infertility treatment can be psychologically burdensome. Evidence on music listening during artificial insemination by husband (AIH) remains limited. We evaluated whether a standardized periprocedural music intervention reduced psychological distress and explored its association with clinical pregnancy.

**Methods:**

In this single-center, open-label randomized trial, 254 women undergoing AIH were allocated 1:1 to routine care or routine care plus two 30-minute music-listening sessions, immediately before and after AIH. The prespecified primary outcome was the continuous 21-item Depression Anxiety Stress Scales (DASS-21) score at follow-up; clinical pregnancy was secondary. DASS-21 was completed at baseline and at day 35 after AIH. Clinical pregnancy was assessed by ultrasonography at days 30 to 35.

**Results:**

Continuous DASS-21 anxiety, depression, and stress scores did not differ between groups after adjustment for baseline scores (all *P* ≥ .868). Absolute postintervention differences were 0.01 point or less for anxiety and depression and 0.08 point for stress, indicating neither statistical nor clinically meaningful improvement. Clinical pregnancy occurred in 34/127 participants (26.77%) in the intervention group and 14/127 (11.02%) in the control group (exploratory secondary outcome; *P* = .001).

**Conclusion:**

The intervention did not improve the primary psychological outcome. The higher clinical pregnancy rate is hypothesis-generating because the trial was not powered for pregnancy, the proposed psychological mechanism was not demonstrated, and nonspecific attention and other unmeasured factors cannot be excluded. Replication using an attention-matched control, immediate state-anxiety and physiological measures, participant-preference assessment, and live-birth follow-up is required.

## 1. Introduction

Infertility is a common reproductive disorder with medical, psychosocial, and societal consequences. Artificial insemination by husband (AIH), clinically corresponding to homologous intrauterine insemination in appropriate couples, remains a first-line option because it is relatively simple, minimally invasive, and less costly than more advanced assisted reproductive treatments.^[1,2]^ AIH outcomes are influenced by biological and treatment-related prognostic factors, including female age, ovarian response, follicular development, endometrial characteristics, thyroid autoimmunity, and sperm functional parameters.^[3–5]^ These biological determinants establish the clinical context for AIH but do not, by themselves, demonstrate an effect of behavioral or supportive care on pregnancy.

Supportive care is relevant for a separate reason: women receiving infertility care commonly report uncertainty, treatment-related anxiety, low mood, and reduced quality of life, which may affect their treatment experience and willingness to continue care.^[6,7]^ Thus, psychological support is justified primarily as patient-centered care; whether it changes reproductive outcomes is a distinct empirical question.

Music-based interventions have reduced short-term anxiety in some procedural settings, including a recent randomized trial during intrauterine insemination, although effects vary across protocols and outcomes. A plausible but untested framework is that auditory features and personal appraisal influence emotion- and arousal-related neural networks. Tempo and rhythmic structure can accompany cardiorespiratory changes, while music-evoked emotion engages limbic and paralimbic regions involved in salience, reward, and autonomic regulation.^[8,9]^ These observations do not establish a fertility mechanism. They instead support testing proximal outcomes such as state anxiety, subjective comfort, heart-rate variability, or cortisol. In the present trial, only the 21-item Depression Anxiety Stress Scales (DASS-21) was measured, and it was assessed weeks rather than minutes after the intervention; consequently, neural, endocrine, and autonomic pathways were not directly tested.

We therefore compared standardized periprocedural music listening plus routine care with routine care alone in women undergoing AIH. The prespecified primary hypothesis was that the intervention would reduce continuous DASS-21 scores. Clinical pregnancy was a secondary, exploratory outcome.

## 2. Methods

### 2.1. Study design and ethical approval

This study was approved by the Ethics Committee of the Fourth Affiliated Hospital of the School of Medicine and International School of Medicine, International Institutes of Medicine. This study was designed as a single-center, open-label, randomized controlled trial conducted at the Reproductive Medicine Center of the Fourth Affiliated Hospital, Zhejiang University School of Medicine, between August 2025 and January 2026. The study aimed to evaluate the effects of music therapy as an adjunct to routine care on psychological distress and clinical pregnancy outcomes in women undergoing AIH. The study protocol was approved by the Ethics Committee of the Fourth Affiliated Hospital, Zhejiang University School of Medicine (Approval No. K205183) and was registered at ClinicalTrials.gov (NCT07356830). Written informed consent was obtained from all participants before enrollment.

### 2.2. Participants

Eligible participants were infertile women scheduled to undergo AIH at the study center. Women were included if they were 20 to 40 years of age, met the clinical indications for AIH, such as ovulatory dysfunction or cervical factors, fulfilled the World Health Organization diagnostic criteria for infertility, defined as failure to achieve pregnancy after at least 1 year of regular unprotected intercourse, were conscious and able to communicate normally, and were willing to participate in the study with written informed consent. Women were excluded if they had medical conditions contraindicating pregnancy, were unable to complete the questionnaire because of hearing impairment, speech impairment, aphasia, or impaired consciousness, had severe genetic, physical, or psychiatric disorders, or had urogenital tract infection or sexually transmitted disease at the time of enrollment.

### 2.3. Sample size and group allocation

After confirmation of eligibility, participants were assigned a sequential study number from 1 to 254 and were randomly allocated in a 1:1 ratio to the intervention group or the control group. Randomization was performed by an independent researcher who was not involved in intervention delivery or outcome assessment, using the random number generator in SPSS version 31.0. A total of 254 women were enrolled, with 127 participants assigned to each group. Because of the nature of the intervention, blinding of participants and intervention staff was not feasible.

The sample size was calculated using the formula for comparison of 2 independent means under a 2-sided test: N1 = N2 = 2[(Zα + Zβ)σ/δ]^2^. With α set at 0.05 and β at 0.10, the minimum required sample size was estimated to be 186 participants. After allowing for a 20% dropout rate, at least 112 participants were required in each group. To ensure adequate statistical power and improve the stability of the findings, 254 participants were ultimately enrolled, with 127 in each group. The sample size was calculated based on the formula for comparing 2 independent means (DASS-21 scores) with α = 0.05 and β = 0.10. It was not specifically powered for the pregnancy outcome. Therefore, the significant difference in pregnancy rates (*P* = .001) should be interpreted as exploratory and hypothesis-generating, requiring validation in larger, adequately powered trials. We have added this caveat prominently in Sections 4 and 5.

### 2.4. Nursing care and music therapy intervention

Both groups received routine health education, procedural explanation, individualized counseling, and supportive communication from reproductive-medicine nurses.

The intervention group additionally completed a 30-minute music-listening session immediately before AIH and a second 30-minute session immediately after AIH in a quiet treatment room. The preprocedure session was intended to target anticipatory situational arousal during the waiting period; the postprocedure session was intended to provide a standardized recovery period after instrumentation. The symmetric timing also standardized exposure around the procedure. These are protocol rationales rather than mechanisms proven by the present data.

A fixed, prerecorded “Nature Relaxation” piano playlist was presented in the same order at 60 to 70 beats/min and 40 to 50 dB. The protocol used low-intensity, slow-tempo instrumental material to minimize abrupt stimulation and to approximate resting physiological rhythms; however, no acoustic spectrum analysis, electroencephalogram, cortisol, or autonomic measure was obtained. The study records identify the pieces “Forest Rhapsody” and “Moonlight”; recording-level metadata were not retained, so no composer-, frequency-, modulation-, or waveform-specific effect is claimed. The available intervention specification and rationale are provided in Table S1, Supplemental Digital Content 1.

A nurse remained in the room during each intervention session and recorded whether the participant remained and completed the full duration. According to the intervention logs, all 127 allocated participants completed both full sessions; no early termination, equipment failure, volume adjustment outside the specified range, or adverse event was recorded.

The protocol did not ask participants to rate music preference, familiarity, acceptability, or perceived benefit. The nurse provided supportive reassurance during music sessions; the control group received routine support but did not receive a time-matched 30-minute quiet-rest session with equivalent nurse presence. Therefore, attention, expectancy, and protected rest cannot be separated from the specific effect of music.

### 2.5. Data collection and outcome measures

Baseline demographic data and outcome variables were collected using standardized case report forms. Baseline variables included age and educational level. Outcome data included DASS-21 depression, anxiety, and stress scores at baseline and follow-up, as well as clinical pregnancy status after AIH. All data were entered into a database and checked for accuracy and completeness.

Psychological distress was assessed using the DASS-21. The DASS-21 includes 3 subscales measuring depression, anxiety, and stress, with 7 items in each subscale. Each item is scored from 0 to 3, and the summed score of each subscale is multiplied by 2 to obtain the final score. Higher scores indicate more severe negative emotional symptoms. According to standard cutoff values, each subscale can be categorized into normal, mild, moderate, severe, and extremely severe levels. The questionnaire was completed by the participants themselves under the guidance of trained nurses, who provided standardized instructions and checked the completeness of the responses on site. DASS-21 was completed at baseline and on day 35 after AIH. Clinical pregnancy was assessed by ultrasonography between days 30 and 35 and defined as an intrauterine or ectopic gestational sac. The primary endpoint was continuous DASS-21 score; clinical pregnancy was secondary.

Because ultrasonography could occur before the day-35 DASS-21 assessment, some participants may have known their pregnancy status when completing DASS-21. The exact person-level sequence and whether results had been disclosed were not recorded. Follow-up DASS-21, therefore, cannot be interpreted as an assessment blinded to pregnancy outcome.

### 2.6. Statistical analysis

Statistical analyses were performed using SPSS Statistics, version 31.0 (IBM Corp., Armonk). Continuous variables were expressed as mean ± SD and were compared between groups using the independent-samples *t* test. Categorical variables were expressed as frequencies and percentages and were compared using the *χ*^2^ test. All tests were 2-sided, and a *P* value of <0.05 was considered statistically significant. Since the categorical analysis was exploratory, we did not perform a Bonferroni correction. However, to avoid overinterpretation, we have explicitly restated in Sections 3 and 4 that the categorical findings represent “a favorable trend” rather than a statistically definitive effect, and we placed greater emphasis on the continuous score analysis, which remains the primary statistical basis of the study. In addition, analysis of covariance was performed to compare postintervention DASS-21 scores between the 2 groups, using the corresponding baseline DASS-21 score as a covariate and the group assignment as the fixed factor.

For the clinical pregnancy outcome, both unadjusted effect estimates and multivariable logistic regression analyses were performed because clinical pregnancy was a binary outcome. Clinical pregnancy status was entered as the dependent variable, and treatment group was entered as the main independent variable. Age and educational level were included as covariates in the multivariable model. Adjusted odds ratios with corresponding 95% confidence intervals were reported.

## 3. Results

### 3.1. Baseline characteristics

**Figure 1.**
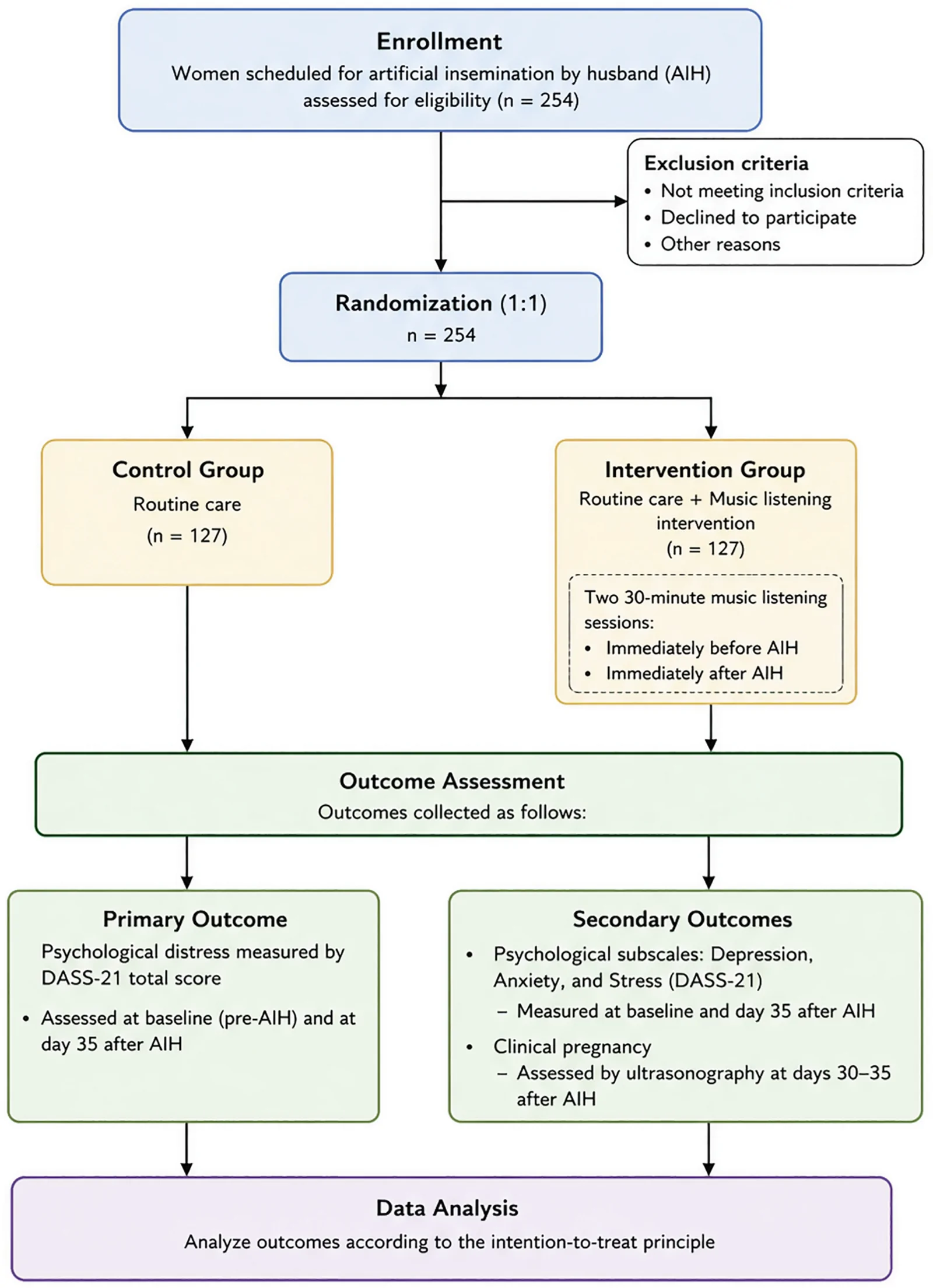
The flowchart of patient enrollment in this study. AIH = artificial insemination by husband; DASS-21 = 21-item Depression Anxiety Stress Scales

A total of 254 women undergoing AIH were enrolled and included in the final analysis, with 127 participants allocated to the control group and 127 to the intervention group (Fig. 1). All participants were married, had experienced infertility for more than 1 year, and fulfilled the clinical indications for AIH. The baseline demographic and clinical characteristics of the study population are summarized in Table 1. In the control group, participants were aged 24 to 38 years, with a mean age of 30.77 ± 3.30 years. In the intervention group, participants were aged 22 to 39 years, with a mean age of 30.74 ± 3.12 years. There was no significant difference in age between the 2 groups (*P* = .469). With respect to educational attainment, 35 women (27.55%) in the control group and 28 women (22.04%) in the intervention group had an educational level below junior college, whereas 92 (72.45%) and 99 (77.96%), respectively, had a junior college degree or above. The distribution of educational level was comparable between the 2 groups (*P* = .310).

**Table 1:** Baseline demographic and clinical characteristics of the study participants.

Variable	Control group (n = 127)	Intervention group (n = 127)	Test statisticsc	*P* value
Age (yr)	30.77 ± 3.30	30.74 ± 3.12	*t* = 0.074	.469
Age range (yr)	24–38	22–39	–	–
Married status, n (%)	127 (100.00)	127 (100.00)	–	–
Infertility duration > 1 yr, n (%)	127 (100.00)	127 (100.00)	–	–
Met AIH indications, n (%)	127 (100.00)	127 (100.00)	–	–
Educational level, n (%)			*χ*^2^ = 1.031	.310
Below junior college	35 (27.55)	28 (22.04)		
Junior college or above	92 (72.45)	99 (77.96)		

AIH = artificial insemination by husband.

### 3.2. Comparison of DASS-21 scores between the 2 groups

Psychological distress was assessed using the 21-item DASS-21, including the anxiety, depression, and stress subscales. As shown in Table 2, no statistically significant between-group differences were observed at baseline for any of the 3 dimensions. The baseline anxiety score was 2.85 ± 3.85 in the control group and 3.41 ± 5.00 in the intervention group (*t* = −1.010, *P* = .157). The baseline depression score was 2.93 ± 5.13 in the control group and 3.03 ± 4.05 in the intervention group (*t* = −0.086, *P* = .466). The baseline stress score was 4.92 ± 5.69 in the control group and 5.22 ± 6.23 in the intervention group (*t* = −0.399, *P* = .345). After the intervention, the mean scores for anxiety, depression, and stress were all numerically lower in the intervention group than in the control group, although the between-group differences did not reach statistical significance. Specifically, the postintervention anxiety score was 1.30 ± 2.88 in the control group and 1.29 ± 3.12 in the intervention group (*t* = 0.042, *P* = .483). The postintervention depression score was 1.44 ± 2.83 in the control group and 1.44 ± 3.87 in the intervention group (*t* = 0.000, *P* = .500). The corresponding stress score was 2.36 ± 3.63 in the control group and 2.28 ± 3.85 in the intervention group (*t* = −0.167, *P* = .434). To further adjust for baseline psychological scores, analysis of covariance was performed. The results were consistent with the independent *t*-test findings: no statistically significant between-group differences were observed for any of the 3 domains (anxiety: *F* = 0.002, *P* = .963; depression: *F* = 0.001, *P* = .994; stress: *F* = 0.028, *P* = .868).

**Table 2:** Comparison of DASS-21 scores between the 2 groups.

Outcome	Time point	Control group (n = 127)	Intervention group (n = 127)	Mean difference, intervention − control (95% CI)	Cohen’s *d*	Test statistic	*P* value
Anxiety score	Baseline	2.85 ± 3.85	3.41 ± 5.00	0.56 (−0.54 to 1.66)	0.13	*t* = −1.010	.318
	Postintervention	1.30 ± 2.88	1.29 ± 3.12	−0.01 (−0.75 to 0.73)	−0.00	*t* = 0.042	.979
Depression score	Baseline	2.93 ± 5.13	3.03 ± 4.05	0.10 (−1.04 to 1.24)	0.02	*t* = −0.086	.863
	Postintervention	1.44 ± 2.83	1.44 ± 3.87	0.00 (−0.84 to 0.84)	0.00	*t* = 0.000	.994
Stress score	Baseline	4.92 ± 5.69	5.22 ± 6.23	0.30 (−1.17 to 1.77)	0.05	*t* = −0.399	.689
	Postintervention	2.36 ± 3.63	2.28 ± 3.85	−0.08 (−1.00 to 0.84)	−0.02	*t* = −0.167	.865

CI = confidence interval, DASS-21 = 21-item Depression Anxiety Stress Scales.

### 3.3. Changes in DASS-21 severity category distribution in the intervention group

To further evaluate the effect of music therapy on psychological status, the categorical distribution of DASS-21 severity levels in the intervention group was examined before and after the intervention (Table 3). After the intervention, the proportion of participants classified as normal increased across all 3 DASS-21 domains. For anxiety, the proportion of participants in the normal category increased from 84% before the intervention to 94% after the intervention. The proportions of participants with mild, moderate, and severe anxiety decreased after the intervention, whereas the proportion with extremely severe anxiety remained unchanged at 1%. For depression, the proportion classified as normal increased from 89% to 98%, while the proportions of participants with mild, moderate, and severe depression declined after the intervention. For stress, the proportion of participants in the normal range increased from 96% to 99%, and no participants were classified as having mild or moderate stress after the intervention. Only 1 participant remained in the severe stress category after treatment.

**Table 3:** Distribution of DASS-21 severity categories in the intervention group before and after the intervention.

Severity category	Anxiety, preintervention, n (%)	Anxiety, postintervention, n (%)	Depression, preintervention, n (%)	Depression, postintervention, n (%)	Stress, preintervention, n (%)	Stress, postintervention, n (%)
Normal	107 (84)	120 (94)	113 (89)	125 (98)	121 (96)	126 (99)
Mild	10 (8)	1 (1)	9 (7)	0	3 (2)	0
Moderate	7 (5)	5 (4)	3 (2)	1 (1)	0	0
Severe	2 (2)	0	2 (2)	1 (1)	3 (2)	1 (1)
Extremely severe	1 (1)	1 (1)	0	0	0	0

DASS-21 = 21-item Depression Anxiety Stress Scales.

### 3.4. Clinical pregnancy outcome

The comparison of clinical pregnancy outcomes between the 2 groups is shown in Table 4. Clinical pregnancy was achieved in 14 of 127 participants (11.02%) in the control group and in 34 of 127 participants (26.77%) in the intervention group. The clinical pregnancy rate in the intervention group was significantly higher than that in the control group, and the difference was statistically significant (*χ*^2^ = 10.538, *P* = .001).

**Table 4:** Comparison of clinical pregnancy rate between the 2 groups.

Outcome	Control group (n = 127)	Intervention group (n = 127)	RR (95% CI)	Odds ratio	Adjusted odds ratio	*P* value
Clinical pregnancy, n (%)	14 (11.02)	34 (26.77)	2.43 (1.37–4.30)	2.95 (1.49–5.83)	2.85 (95% CI: 1.53–5.68)	.001

CI = confidence interval, RR = risk ratio.

## 4. Discussion

The principal finding is that the music intervention did not improve the prespecified primary outcome. Continuous anxiety, depression, and stress scores were statistically indistinguishable between groups, and the absolute postintervention differences were clinically negligible. The primary hypothesis of an effect on DASS-21-measured psychological distress, therefore, failed in this trial.

Several explanations are plausible. First, baseline scores were low, and most participants were already in the normal range, leaving little room for improvement (floor effect). Second, DASS-21 measures symptoms over the preceding week and was administered 35 days after 2 brief periprocedural sessions; it is poorly aligned with a putative immediate change in state anxiety or arousal. Third, both groups received supportive communication, which may have reduced distress in both arms. Fourth, the intervention was standardized rather than preference-based, and preference, familiarity, dislike, and perceived benefit were not measured. Finally, the trial may have been powered for the assumed effect used in planning but not for the very small effect actually observed. The confidence intervals and near-zero point estimates make a clinically important effect on continuous DASS-21 unlikely under this protocol.

Knowledge of pregnancy status is an additional interpretive problem. Ultrasound was performed between days 30 and 35, whereas DASS-21 was fixed at day 35. Some participants may, therefore, have completed the questionnaire after learning the result. Pregnancy knowledge could lower or increase distress and may obscure or create group differences unrelated to the intervention. Because individual sequencing and disclosure were not recorded, the direction and magnitude of this bias cannot be determined.^[10,11]^

The absence of statistically significant between-group differences in continuous DASS-21 scores should be interpreted cautiously, rather than as evidence of no psychological effect. In the present cohort, baseline mean scores for anxiety, depression, and stress were low in both groups, and the majority of participants were already within the normal range before intervention. This pattern suggests a floor effect, whereby the scope for further numerical reduction was limited. A recent mixed-methods study in Chinese women undergoing assisted reproductive treatment reported that psychological burden is common but heterogeneous, with anxiety more prevalent than depression or stress, and with symptom severity influenced by treatment-related uncertainty, family support, work conflict, and financial burden. Under such circumstances, brief supportive interventions may improve patient experience without necessarily producing large changes in continuous psychometric scores across an entire sample.^[12]^ The categorical findings in the intervention group provide additional context for interpreting the DASS-21 results. After music therapy, the proportion of participants classified as normal increased across all 3 DASS-21 domains, particularly for anxiety and depression. This pattern indicates that the intervention may have helped move a subset of participants from mild or moderate symptom levels into the normal range, even though the overall mean differences remained small. Recent evidence suggests that psychological interventions in infertile women can improve anxiety, depression, stress, infertility-specific distress, and well-being, but that effect sizes and the apparent superiority of any single modality are variable across studies because of differences in intervention content, intensity, treatment stage, and outcome measurement. Thus, it is not unexpected that some participants moved from mild or moderate symptoms into the normal range, while the overall mean DASS-21 scores changed only slightly between groups.^[13,14]^ Music (particularly at 60–80 bpm) may modulate the hypothalamic–pituitary–adrenal axis via auditory pathways, potentially reducing salivary cortisol levels and enhancing parasympathetic activity (as reflected by increased heart rate variability). During AIH, such acute physiological regulation could theoretically improve uterine blood flow and reduce cervical tone, creating a more favorable environment for sperm transport. However, these mechanisms remain speculative and were not directly measured in our study. Future research incorporating biomarkers (e.g., cortisol, autonomic measures) is needed to validate these pathways.

The current findings are broadly consistent with emerging literature on music-based interventions in reproductive and procedural care. A 2025 randomized controlled trial of women undergoing intrauterine insemination reported that music exposure during the procedure reduced postprocedure anxiety and was associated with high patient satisfaction, although it did not report pregnancy outcomes. Similarly, a 2025 study of women undergoing gynecological and fertility procedures found that music appeared acceptable and useful for reducing anxiety in this context.^[10]^ Outside reproductive medicine, recent reviews have also concluded that music intervention is a promising nonpharmacological strategy for reducing preprocedural or perioperative anxiety, although heterogeneity in implementation and reporting remains substantial. These data support the view that music can improve the short-term emotional response to invasive or stress-inducing reproductive procedures, and they place the present AIH findings within an increasingly coherent evidence base.^[15,16]^ Importantly, the effect-size estimates indicated that the intervention was associated with an absolute increase of 15.75 percentage points in clinical pregnancy rate, corresponding to a 2.43-fold higher relative probability of clinical pregnancy and 2.95-fold higher odds of clinical pregnancy compared with routine care alone.

The most clinically notable result of the present study was the significantly higher clinical pregnancy rate in the intervention group. This finding is of particular interest because prior evidence in assisted reproduction has suggested the potential benefit of music therapy for patient experience and anxiety, whereas effects on pregnancy outcomes have been less certain. The 2025 randomized intrauterine insemination trial demonstrated an anxiety benefit but did not evaluate conception outcomes, and earlier evidence summarized in subsequent reviews suggested that improvements in pregnancy rates, when observed, were not consistently significant. Our results therefore extend the current literature by indicating that a brief music-based intervention administered around AIH may be associated with an observed difference in early reproductive outcome. Given the randomized design and baseline comparability of the 2 groups, the observed difference is unlikely to be explained solely by measured baseline demographic imbalance.^[17,18]^

Several mechanisms may account for the divergence between modest psychological-score effects and a significant reproductive effect, although these mechanisms remain inferential. Music may attenuate acute situational anxiety during key treatment windows more effectively than it changes global symptom scores assessed over the preceding week. This distinction is important because transient procedural stress may influence muscular tension, autonomic balance, pain perception, procedural tolerance, and overall treatment experience, even when longer-interval questionnaire scores change little. Recent reviews of technology-based and perioperative music interventions have similarly suggested that music may act primarily through short-term modulation of anxiety, arousal, and procedural comfort, with implementation characteristics such as timing, repetition, and patient preference potentially affecting efficacy. Thus, it is plausible that music therapy improved immediate periprocedural regulation in ways that were clinically relevant to AIH success but only incompletely reflected in posttreatment DASS-21 means.^[19–21]^

These findings have several clinical implications. Music therapy is noninvasive, inexpensive, and operationally simple, and it can be integrated into nursing workflows without interfering with standard reproductive procedures. Recent reviews in procedural medicine indicate that music-based interventions are generally feasible and acceptable, particularly when delivered through practical, low-burden formats. In the fertility setting, where emotional distress may arise from uncertainty, repeated treatment exposure, and sociocultural pressure, even modest improvements in patient comfort may be clinically relevant. The present data suggest that incorporating music therapy into routine AIH care may be reasonable, especially for women with elevated treatment-related anxiety or previous unsuccessful cycles. However, these results should be implemented with appropriate caution until replicated in larger multicenter studies. This study also has limitations. First, it was conducted at a single center, which may limit generalizability. Second, the trial was open label; therefore, expectancy effects and performance bias cannot be excluded. Third, the intervention period was short, and the protocol did not evaluate dose-response relationships related to music type, session frequency, patient preference, or cumulative exposure. Recent reviews have noted that variability in intervention implementation is a major source of heterogeneity in music-intervention research. Fourth, DASS-21 is a self-reported scale and may be less sensitive to subtle short-term procedural changes than instruments focused on state anxiety at the moment of treatment. Fifth, the study assessed clinical pregnancy rather than ongoing pregnancy or live birth, so the longer-term reproductive relevance of the observed benefit remains uncertain. Sixth, the study was not powered or prespecified for subgroup analyses; therefore, we cannot reliably determine whether the intervention effect varied by baseline distress level or treatment history. Finally, we also cannot rule out residual confounding. Factors not collected in this study, such as the cause of infertility, ovarian stimulation regimen, previous AIH attempts, partner support, and financial or social pressure, may have affected both emotional status and pregnancy outcome. Owing to the open-label design, we cannot exclude the possibility that placebo effects or positive expectancy (i.e., participants’ belief that music would help) contributed to the observed differences in pregnancy outcomes. Future studies might incorporate an active control group, such as quiet rest or neutral auditory stimuli, to isolate the specific effect of music.

## 5. Conclusion

In women undergoing AIH, adjunctive music therapy was associated with a favorable trend in psychological severity categories and an exploratory finding of a higher clinical pregnancy rate. However, since pregnancy was a secondary endpoint and the sample size was calculated based on psychological scores, these reproductive results should be considered preliminary and hypothesis-generating and require confirmation in dedicated, adequately powered multicenter trials.

## Author contributions

**Conceptualization:** Xianwen Jin, Rubing Hu, Xiaocha Zhu, Xuewei Chen.

**Data curation:** Xianwen Jin, Rubing Hu, Xiaocha Zhu, Xuewei Chen.

**Formal analysis:** Xianwen Jin, Rubing Hu, Xiaocha Zhu, Xuewei Chen.

**Funding acquisition:** Xianwen Jin.

**Investigation:** Xianwen Jin.

**Writing – original draft:** Xianwen Jin.

**Writing – review and editing:** Xianwen Jin.


