# Supplementary material for: Music therapy in artificial insemination by husband: Effects on psychological distress and clinical pregnancy rate
Source: Medicine (Baltimore). 2026 Sep 11;105(37):e50655. doi: 10.1097/MD.0000000000050655 (PMC13574481; doi:10.1097/MD.0000000000050655)
Supplement: Supplementary file 1 [file medi-105-e50655-s001.docx]

**Supplementary Table S1. Available intervention specification and selection rationale**

| **Component** | **Recorded specification** | **Rationale and interpretive boundary** |
| --- | --- | --- |
| **Program** | Fixed pre-recorded “Nature Relaxation” piano playlist; same order for all participants | Standardized exposure; did not test individualized preference. |
| **Identifiable titles** | “Forest Rhapsody”; “Moonlight” | These are the titles recoverable from study records. Composer, performer, edition, file identifier, duration, key, and spectral profile were not retained; no recording-specific neurobiological claim is made. |
| **Tempo** | 60–70 beats/min | Chosen as a slow, resting-range tempo intended to avoid high arousal; tempo does not guarantee a uniform emotional response. |
| **Sound level** | 40–50 dB | Comfortable low-to-moderate listening level; individual ear-level exposure and frequency spectrum were not measured. |
| **Texture** | Instrumental piano/nature-relaxation style | Selected to limit lyrical or semantic distraction; cultural familiarity and preference were not assessed. |
| **Dose and timing** | 30 min immediately before and 30 min immediately after AIH | Targets anticipatory waiting and immediate recovery; no proximal state-anxiety or physiological outcome was measured. |
| **Delivery** | Quiet room; nurse present; identical order | Improved consistency but introduced additional attention and protected-rest exposure relative to control. |

*Abbreviation: AIH, artificial insemination by husband.*
